# Indigenous astronomical knowledge based seasonal weather forecast: evidence from Borana Oromo pastoralists of Southern Ethiopia

**DOI:** 10.12688/f1000research.121926.1

**Published:** 2022-10-25

**Authors:** Abera Bekele Dinsa, Feyera Senbeta Wakjira, Ermias Teferi Demmesie, Tamirat Tefera Negash

**Affiliations:** 1 College of development studies, Addis Ababa University, Addis Ababa, 20120, Ethiopia

**Keywords:** Astronomy Moon, Borana Oromo, Ethiopia, Weather Forecast, Indigenous Knowledge

## Abstract

Indigenous knowledge is still widely used by communities around the world to overcome social-ecological challenges. Borana Oromo pastoralists of Southern Ethiopia have been searching for future weather phenomena using their indigenous knowledge. This study examines indigenous knowledge-based seasonal weather forecasts through using observable physical and temporal patterns of astronomic objects. Data were generated through using focus group discussion, experimental knowledgeable groups and direct observation in the year 2021. The finding reveals that Borana Oromo pastoralists commonly define physical and temporal outlooks of the Moon that are changed and/ or displayed periodically within a range of varying contexts. The observation of the Moon features have been offering sufficient lapse time in a more than/within ahead of a season/ and is relatively more accurate than the other traditional forecasting objects. The study showed that both temporal and spatial dimensions of the forecasting were actually happened in *Ganna* (March to May rainfall), *Adolessa* dry season (June to September) and *Hgayya* rainfall (October to November) seasons of 2021. This indigenous weather forecasting practices are still regularly used in every livelihood decision making process. However, this indigenous weather knowledge of Borana Oromo pastoralists of Southern Ethiopia is not yet mainstreamed in the formal institutional structures. Curtailments of mobility, severity of drought, weakening of traditional institution, leaving of traditional life style and death of the knowledgeable elders are the major factors that are challenging the potential effects of the indigenous weather forecasting indicators of the features of moon in the study area.

## 1. Introduction

Indigenous knowledge (IK) is not a new concept ( UNFCCC, 2013; Chand *et al.*, 2014; Ibrahimm, 2020); it has been in existence ‘since humankind’ ( Sillitoe and Sillitoe 2006; Bruchac, 2020). However, ( World Bank, 2009; Tang, 2012) noted that IK is a wisdom that is unique to a given culture/society with in a given geographical area. In line with this, ( SsetteeE, 2007; Fernandez, 2015) argued that IK is an essential survival mechanism: it is the sum total of knowledge and practices based on people’s accumulated experience in dealing with the problems related to all aspects of life system. In this regard, IK practices are not static traditions ( Langill, 1999; SsetteeE, 2007; MoSTE, 2015); it is rather complex, holistic, dynamic/changing through adaptive responses to external and internal changes/shocks and have evolved throughout the generations from wisdom distilled in trial and error ( Eyong, 2001; Mandaluyong & Donato-kinomis, 2016; Rahman & Rusli, 2017; Ibrahimm, 2020). These characteristics of IK are the driver in formulating cost-effective and participatory adaptation measures/to prepare and respond better to disaster risks ( Theodory, 2016; Berkes *et al.*, 2021). The logic behind this is that ‘intrinsic resilience’ is the outcome of indigenous adaptation practices ( Kirmayer *et al.*, 2011; Hiwasaki, 2014; Rustomjee, 2015).

In connection with this ( Fernandez, 2015; MoSTE, 2015; Mandayulong *et al.*, 2016; Nakashima *et al.*, 2018), noted that the ancient human civilization/knowledge, technology and practices that are distilled through millennia of experimentations including fire making, domestication of animals and plants, knowledge on cultivation/husbandry, celestial knowledge and etc … which have still multi-dimensional effects on human wellbeing are rooted in IK. Even, in the age of the current modernization/globalization period, IK is functional in all fields of humanity and sustainability science in the forms of literary knowledge, languages, belief, arts, music, science, agriculture, ecological knowledge, engineering and governance, etc. ( Eyong, 2001; Mandaluyong & Donato-kinomis, 2016; Lambert & Scott, 2019). Studies assert that IK has value, not only for the culture in which it evolves, but also for those who are striving to improve conditions in rural livelihoods ( Apgar & Apgar, 2010; Nakashima *et al.*, 2018; Panampitiya, 2019). Hence, IK cannot be separated into discrete disciplinary departments and time.

Puffer & Associate (1995), Session *et al.* (2006), Mauro *et al.* (2010), Lunga (2014) explored that marginalization of the IK worldview was underway since the 12 ^th^ century with the increasing dominance of the modernization values/cultures. In addition, lack of documentation, influence of modernization, departing of IK based life style and/or death are among the factors that are challenging IK ( Saskato, 2010; Apgar & Apgar, 2010; IASG, 2014). However, recently, the role of IK in development is more recognized at all levels in response to increasing multi-hazard/disaster, climate variability and change and as a response to failure of the blue print development planning ( UNFCCC, 2013; MoSTE, 2015; Theodory, 2016; David-Chavez, 2019).

Historically, prediction of seasonal weather and impending disasters have been an integral part of indigenous peoples’ adaptation and resilient strategies which are used to interpret observed natural signals to predict future weather patterns ( Rahman & Rusli, 2017; Mosime, 2018; Šakić, 2020; Nyadzi *et al.*, 2021). Still today, indigenous communities in different parts of the world have relied on IK weather forecasting in making agricultural practices, environmental conservation, governance, in disaster risk reduction measures; and to deal with continuum of dynamic aspects of social, political, economic, environmental and technological change in resilience process ( Tang, 2012; Ibañez, 2014; Mandayulong *et al.*, 2016; Nakashima, *et al.*, 2018).

Indigenous astronomy is about people’s relationships with the sky, not just about the sky ( Willis & Ii, 2011; Lawrence, 2021a). Important aspect of indigenous astronomy is how knowledge about the sky is culturally encoded weather in every daily livelihood decision making ( Scofield, 2010; Ruggles, 2015; Lee *et al.*, 2020). Studies posited that even today, the use of indigenous astronomical seasonal weather forecasting information is functional in Africa ( Angchok & Dubey, 2006; Holbrook, 2016; Mafongoya and Ajayi, 2017; Chang *et al.*, 2019). In Uganda, Karamoja communities used star relationship with the moon to forecast future weather events ( Okonya & Kroschel, 2013; Oruru *et al.*, 2016). Holbrook, (2016) stated that in South Africa indigenous knowledge of changing seasons, lunar cycle, shape, location of the Moon and the stars are used to predict drought. In Zimbabwe, a set of the biotic and abiotic weather forecasting objects are used for agricultural practices ( Jiri *et al.*, 2015). Studies in Burkina Faso and Lesotho quoted in ( Ifejika *et al.*, 2009) examined that local forecasting knowledge seems to be less widely used than in the past.

Apparently, in rural areas of Ethiopia indigenous knowledge practices are still sustained/dominated in all aspects of life and livelihoods making ( Workineh *et al.*, 2010; Ibinarriaga, 2020). In connection with this, Borana pastoralists define outlooks of astronomic objects, observe atmospheric characteristics, read physical condition of biotic objects and rememorize the unique and known weather events in each *Geda* cycle, periodically in the process of future weather forecasting. However, studies and proper documentation have not yet been undertaken to preserve this invaluable indigenous weather indicating knowledge and practices of the Borana Oromo pastoralists. In addition, no area specific study is undertaken on practical experiences of community-based approaches vis-à-vis weather indicating features of the astronomic objects including moon in the study area. Hence, the objective of this paper is to examine indigenous astronomic seasonal weather forecasting knowledge and practices of Borana Oromo pastoralists of southern Ethiopia with insights on the weather indicating features of the Moon. This study is tried to answers three interrelated questions. 1) What are the future weather indicating features of the Moon in the study area?, 2) Is there sufficient lapse time between forecasting and occurrence of the weather events?, 3) Are indigenous weather forecasting practices currently being used by the study community in the process of the livelihood decision making?

### 1.1 Concept and definition of indigenous knowledge

Cognizant with the above narratives, several scholars have developed definitions of indigenous knowledge in respect of the aspect/contexts of the indigeneity matters consistent with the premise of the disciplines under concern. Hence, it is beyond the theme of this study to thoroughly deliver/articulate all existing debates in the literature. In this regard, owing to the fact that a given IK is embedded/is the reflection of a society within a given specific environmental/geographical area ( Tang, 2012); it is better to highlight the definition of the phrase indigenous science and indigenous peoples separately before articulating the definition of the IK to more substantiate the topic under the study. In connection with this, it is believed that Indigenous science and indigenous peoples are the subset of the indigenous knowledge/both are encrusted in IK ( Eyong, 2001; Lunga, 2014; Lambert & Scott, 2019). The existing literature classifies indigenous sciences as individual and social/cultural level definitions. In this regard, this study highlights on the social/cultural level, definitions which define indigenous science/ethno science as a culture/society dependent collective rational perceiving of a reality ( Theodory, 2016; David-Chavez, 2019). Indigenous science classifies the objects, activities, and events within a given universe. Indigenous science interprets the local world cultural perspective of indigenous agriculture, astronomy, navigation, mathematics, medical practices, engineering, military science, architecture, and ecology, *etc.* ( Scofield, 2010; Siseho, 2013; MoSTE, 2015; Holbrook, 2016; Panampitiya, 2019; Ibinarriaga, 2020). In addition, processes of science that include rational observation of natural events, classification, and problem solving are woven into all aspects of indigenous science ( Langill, 1999; Rahman & Rusli, 2017; David-Chavez, 2019).

Apparently, it is also beyond the scope of this research to deliver the details of the social science disciplines perspectives scholars’ debate on the issues of the indigenous peoples. In line with this study, it is more applicable to use/adopt the development perspectives definition of the UNPFII studies cited in ( UNFCCC, 2013; Rahman & Rusli, 2017; Šakić, 2020) and the World Bank studies cited in ( Langill, 1999; Eyong, 2001; SsetteeE, 2007; Burgos-Ayala *et al.*, 2020) that defines indigenous peoples as groups of native inhabitants having their own territory, languages, political system and self-rule, have historical continuity with their neighbors pre-settlers societies, with distinct social and cultural identity; resolve to maintain and reproduce their ancestral environments which are vulnerable/disadvantaged in the development process aftermath of the intrusion of the external cultures/actors. In connection with this ( Rajasekaran, 1993; Panampitiya, 2019; Bruchac, 2020), posited that worldwide indigenous peoples speak ‘two-thirds of humankinds’ more than 7,000 known spoken languages and many of these languages are, currently, under use and/or spoken by very few peoples; it is expected that unless protection is maintained, 70 to 90 per cent of these languages will be lost by 2115.

In regard with this, before inception of defining the phrase indigenous knowledge having the highlight over view of the terms knowledge could help to understand the brief context of the IK. In line with this ( Tang, 2012; IASG, 2014; Ibañez, 2014; Theodory, 2016; Aderemi, 2017; Mafongoya and Ajayi, 2017; Šakić, 2020), stated that knowledge is the awareness or understanding of a practical or theoretical thing or fact. It further stated that knowledge embraces knowledge of tools and techniques for assessment, acquisition, transformation, and utilization of resources in the locality ( SsetteeE, 2007; Ibañez, 2014; Fernandez, 2015; Mandaluyong & Donato-kinomis, 2016; Ajagbe, 2018; Williams & Sikutshwa, 2020) also noted that it is indigenous because it differs from known forms of formal knowledge (scientific, Western, modern) in the contextual sense (as IK is deeply rooted in its environment, history, and new experiences) and the epistemological nature of IK is holistic. This kind of knowledge remains the information base for a society, which facilitates communication and livelihood decision-making ( Apgar & Apgar, 2010; Nakashima *et al.*, 2018; David-Chavez, 2019) attempted to isolate indigenous science from its holistic body of knowledge and reveals that indigenous knowledge bears both scientific and technological threads but in its creation and use it is simply practical/pragmatic knowledge and not ordinarily identified as a science. Thus, understanding and analysis of indigenous science tends to be done with reference to well-established Western modern science (WMS), which people are already familiar with ( SsetteeE, 2007; Fernandez, 2015; Mandaluyong & Donato-kinomis, 2016). In synopsis with this, this study adopted, aggregated and synthesize IK definition of mainly IPBES, World Bank and UNPFII studies cited in ( Langill, 1999; SsetteeE, 2007; Sirima, 2015) which stated that IK is a multifaceted array of area-specific knowledge; know how, practices, representation of identity and survival strategy, adaptive/dynamic with change and holistic in nature, intergenerational, and which guides societies in their interactions with their surrounding environment. Indigenous knowledge can help build resilience in three ways: increasing the range of available knowledge; providing the basis for adaptations; and enabling social practice and learning ( Panampitiya, 2019; Berkes *et al.*, 2021).

### 1.2 Description of the study area and research methodology

Astronomically, theBorana zone is between 3° 31’ 31” to 6° 35’ 37” latitude and 36° 42’ 38” to 39° 45’15” E longitudes ( Homann *et al.*, 2008; Lemenih *et al.*, 2011). It is in the southern part of the Oromia regional state Of Ethiopia. Yabello the capital town of Borana Zone is 570 km South of the capital city, Addis Ababa ( Tura and Reddy, 2015). It borders Kenya in the South, Somali Regional State and Gudji Zone of Oromia in the East, and the Sidama Region, Southern Nations, Nationalities, and Peoples Region (SNNPR) in the north and west ( Godana, 2016). The landscape of the Zone is mainly lowlands with slightly undulating peaks up to 2000 meters above sea level in some areas ( Berhanu and Beyene; 2015). The land area is 63,939 km ^2^ ( Dalle, 2014; Dirriba *et al.*, 2020). The projected population of the Borana Zone is 1,626,930 (male: 821,733; and female: 805,197) with the majority (97%) living in rural areas; Borana Oromo is the largest community in the area with interconnected Oromo groups of Garba and Burdji ( Desta, 2006; Homann *et al.*, 2008; Berhanu, 2011; Demisachew & Abiyot, 2019).

**Figure 1.**
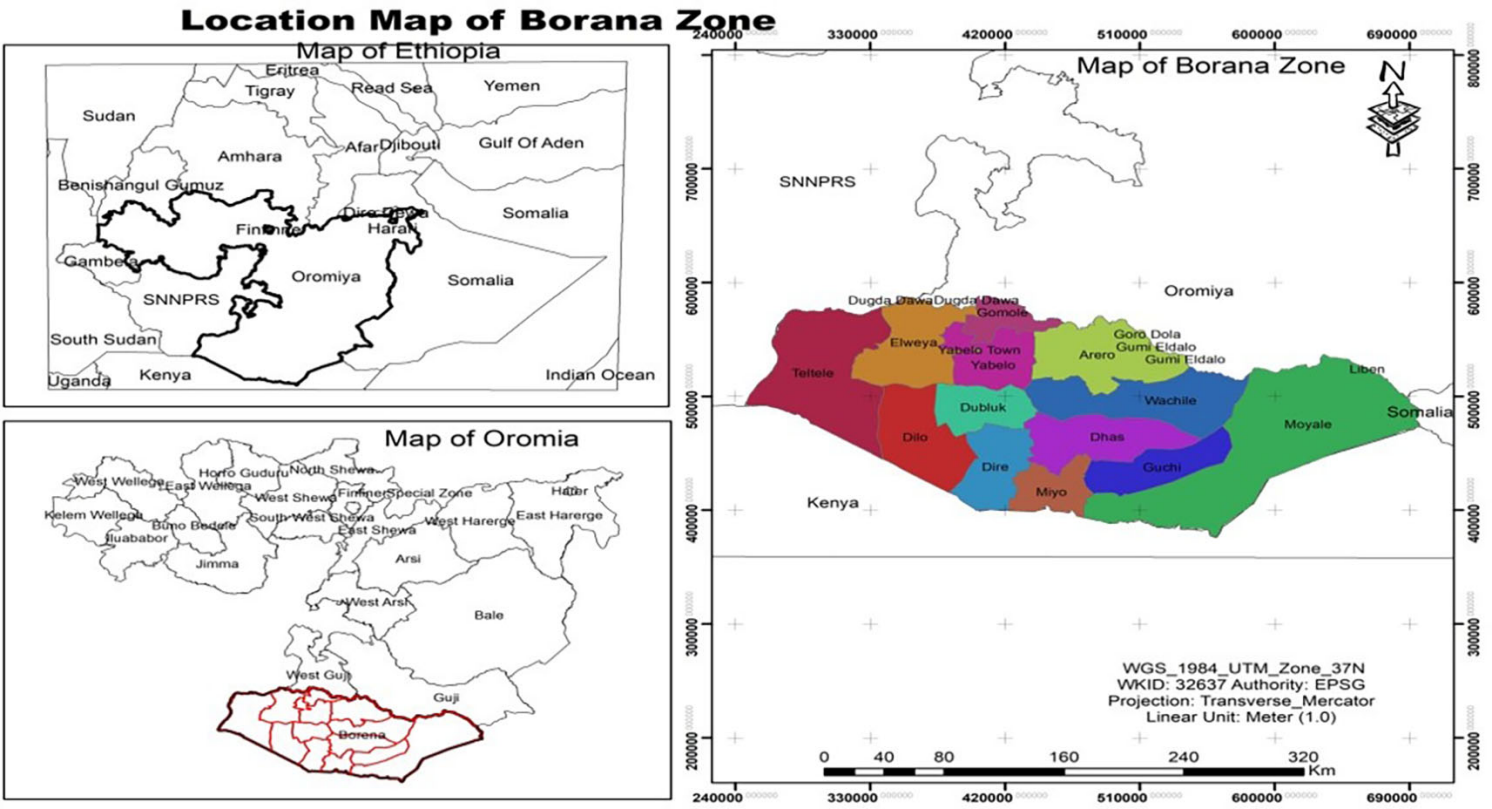
Location map of the study area. Source: OPC, 2021.

Agro-ecologically, it is semi-arid lowlands and frequently prone to devastating severe drought with an increasing trends ( Ambelu, 2015). There are four locally defined seasons in which annual rainfall distribution and the dry period patterns of the study area are bimodal in character ( Gebissa Takele, 2015). Mean annual rainfall of the area is between 400 to 700 mm ( Berhanu, 2011). In the normal scenario, the long/main rain season ( *Ganna* season rainfall) is between March and May; and the short rain season ( *Hagayya* rainfall) is between September and November ( Lemenih *et al.*, 2011; Zewdie *et al.*, 2015). The onset and cessation of both rainfall seasons are often irregular in duration; and are scattered in spatial coverage ( Bule, 2021). The warm dry season ( *Bona Hagayya*) is between December and February with high evapo-transpiration rate ( Dalle, 2014); and the cool dry season ( *Bona Adoolessa*) is between June and August ( Lemenih *et al.*, 2011).

The main important sources of livelihoods are pastoralism and/or livestock production; under semi-sedentary basis ( Tadicha, 2015). Borana pastoralists are known worldwide due to the famous Boran cattle breeds ( Desta, 2006). A periodical livestock mobility strategy is the common drought risk mitigation which allow Borana pastoralists to use ecologically/seasonally variable scarce communal natural resources ( Mengistu, 2015). The holistic Oromo traditional administration, the *Geda* system, is still mainly functional among the Borana Oromo community of the Southern Ethiopia. In line with this, traditionally, in the Borana zone of Southern Ethiopia, indigenous weather forecasting knowledge practices and information are the driver/basis of livelihood decision making. Drought, scarcity of water, rangeland degradation, poor market system, lack of infrastructure, weak disaster preparedness, weak service delivery system/lack of strong institution, weakening of the customary indigenous system and lack of pastoral oriented development policy exacerbated the adverse effects of the drought in the area ( Godana, 2016; Mohamed, 2019; Dirriba *et al.*, 2020).

## Methods

All research subjects and variables under the study were purposively selected to directly reach/explore the major representative samples and quality data in the study area. To easily manage the demeanor of this study, 13 districts of the zone were grouped in to five clusters that were consist of Yabello, Teltele, Gomole, Dirre and Moyale which are based on the proximate of the district to each other for the purpose of this study. Primary data were generated through using focus group discussions, experimental groups, knowledgeable informants, and direct observation. In the present study, indigenous knowledge of astronomical seasonal weather representative variables of observable external physical features of the stars were mainly explored using well experienced elders in the community.

Data were collected through using focus group discussion participants, experimental groups and key informant interview participants that were held at the aforementioned clusters. Accordingly, five focus group discussions; each consists of 12 participants (8 males and 4 females) were participated in data generation. Regarding the experimental groups, five groups (clusters) each consists of 4 (a total of 20 participants) well experienced traditional astronomical weather forecasters were used for gathering data. In addition, 10 key knowledgeable informants (6 males and 4 females) were interviewed. During data collection, four local enumerators who have in-depth knowledge of variable under the study were participated in all the steps and phases of the data collection after receiving training on the topic and issues of the study. The first author has more than eight years career experiences and native with the same community in different geographical area. Selection of the participants and formation of the clusters were undertaken in collaboration with the relevant stakeholders/organizations including GOs, NGOs, and community based organization and community representatives.

Data were collected in three months lead time of each season. The data were collected in four subsequent rounds/seasons; starting from the seasons of *Bona Hagaya* (warm dry season of December to February), *Ganna* (main rainfall season of March to May), *Bona Addolessa* (cool dry season of June to August) and *Roba Hagayyaa* (small rainfall season of September to November) of 2021. According to the local calendar of the study area New Year is started on first day of December.

The details of the data were collected through reading physically/spatially and temporally varied multidimensional outlooks of the weather indicating features of the moon. In this regard, defining, reading and observation of the direction/inclination of the new Moon, cloud surrounding the moon/cloud not surrounding the moon and cloud pattern of the moon were done in the data collection process to know the upcoming season’s weather phenomena in the study area. In connection with this, Borana Oromo abstractly made sequential list of 27 traditional day patterns of the moon which are reconfigured/varied in chronological order with in each of the 12 moon/months of the year, moon day/the day on which new moon is re-emerged in the night sky and rainfall patterns of the moon day, *etc.* are used in the data collection process of this research. In this research, during defining, reading and observation; ratification of the projection/forecast (mainly normal occurrence of rainfall/severe drought) was established by the experimental groups/experienced traditional weather forecasters upon the interpretation of the future weather indicating features of the moon.

In connection with this, data were collected in three months lead time of *Bona hagayyaa* (dry season of December to February), *Ganna* rainfall season (main rainfall of March to April), *Bona Addolessa* (cool dry period of June to August) and *Hagaya* rainfall season (September to Mid-November) of 2021. Finally, verifications of the accuracy/occurrence of the future weather phenomena were done against the projection/established forecast of the future weather indicating features of the moon. In this study, only qualitative data were collected through using semi-structured questioners and open ended interview guides. Finally, data were analysed through using Bazeley (2009) methods of qualitative data analysis techniques.

## 2. Results and discussion

It is identified through this study that indigenous weather forecasting knowledge is the fundamental driver of the livelihood decision making among Borana Oromo pastoralists of the Southern Ethiopia, since time immemorial. In this regard, in the process of making this in to effects; traditionally, indigenous Borana Oromo community could define, read, and interprets all objects in the easily observable/biosphere/and hardly accessible environment/Universe/to forecast current particular and future weather events in the process of making/ensuring drought resilience.

### 2.1 Indigenous weather forecasting knowledge and practices of borana oromo pastoralists

It is identified through this study that commonly practiced indigenous based weather forecast indicating objects that are exhibit change naturally with in a continuum of varying temporal/seasonal contexts that are, currently, in use by the study area communities are mainly categorized into astronomic objects, atmospheric characteristics, biotic objects and the *Geda* cycles/historical experiences.

In the process of weather forecasting; Borana Oromo pastoralists could regularly define multidimensional outlooks of the astronomic/celestial objects which are displaying varied indicators/signs with in a context of changing spatial and temporal patterns. In this regard, alignment/defect of the alignment/, direction, group formation, color/physical features, size, ray, type, and duration of the observation of the stars in the sky are used in weather forecasting. In connection with this, Borana pastoralists’ abstract sequential list of day patterns of the emerging of the new moon/moon day/, cloud formation/no cloud formation, inclination of each of the new emerging moon and colour pattern of the moon are defined and interpreted in the indigenous weather forecast process. In addition, ring formation, direction/position, size, partial or full eclipse (death) and color/physical features of the sun are regularly defined to indicate either of the fortune/wickedness of the forthcoming season. In addition, it is identified that they could make the prediction through defining characteristics/conditions of the biotic objects that are varying in alignment with temporal patterns and are influenced by the pulling effects of the tell-connections with in the Geo-space (wind circulation, cloud formation, temperatures and lightening with in the land, ocean and atmosphere) of the their environment. In line with this, pastotalists of the study area are accustomed in reading indicators such as Physical condition, physiological features, movement/flying patterns, rhythm/sound and smell of biotic objects; observing of cloud, wind, temperatures and lightening that are varying vis-a-vis with in the temporal and spatial contexts are used in the study area in weather forecasting. Weather Forecasting is also undertaken through using *Geda* Cycle. There are seven cycles in *Geda* system. Each cycle has completed in eight years and each cycle of the *Geda* has its own distinctive name. Each *Geda* cycle is known by its usual and unique expected weather events which are greatly known in that cycle. Hence, it is possible to forecast weather condition of the coming 40 years through using *Geda* cycles. This is in consistent with the Studies of ( Bruchac, 2020; Zounon *et al.*, 2020) noted that indigenous community in Zambia and Benin use plant, animal, historical experiences and astronomical elements to forecast weather. In Nigeria ( JU 2018) argued IK indicators that are used to predict the severe drought are fruits drying/falling off from trees, increasing occurrence of termites, shedding of tree and appearance of rainbows.

As it is identified through this study, all members of the community have no skill of forecasting upcoming season of weather events from all objects at equal level. In this regard, one do have the skill of forecasting upcoming season of weather events via defining of astronomic objects, others have observe/interprets atmospheric objects/or read physical and physiological condition of biotic objects, etc. Even though, all fathers have shared/transfer/their skill of weather forecasting to all their children, including women without any gender restriction; all the children of a given household/family/do not have capacity, wish and interest to keep all the elements of forecasting signs equally at required level. In connection with this, there are few individuals in the community who are qualified in making accurate forecasting via using a given specific objects in the environment. Individuals those who are qualified in reading internal intestinal structures of livestock are locally called *Uchu*; those who are qualified in defining physical and temporal features of the moon are locally called ‘ *Ayyaantu*; individuals those who are qualified in identifying the star moon-alignment are called *Hedduu* and those individuals who are qualified in rememorizing the known events in each *Geda* cycles are called *Arga-dhageti.* In this regard, in a village there is one trusted forecaster in each of the aforementioned capacity; an aggregate of total experienced forecaster might range up to 1% (one per cent) of the community in the study area. Although it is the driver of the livelihood decision making at the local level; IK weather forecasting of Borana pastoralists were not mainstreamed in the formal institutional structures.

**2.1.1 Weather indicating features of the Moon**

It is identified through this study that, it is a widely common/accustomed/ ordinary traditional practice that any pastoralists in the study areas have regularly searching future outlooks of the seasonal weather phenomena, since time immemorial. In line with this, in the study area; one of the widely used practice of indigenous based weather indicating objects that are exhibit change naturally with in a continuum of varying temporal and/or seasonal and spatial contexts are observable external physical features of the moon. It is identified that Borana Oromo pastoralists regularly define/read physical and temporal outlooks of the moon that are changed and/or displayed periodically with in a range of varying contexts. This is in consistent with the Study in Botswana quoted in Lorreta (2014), posited that it was customary for the ancient people to align their sacred monuments with precise solar, lunar and stellar positions; ( Lee *et al.* 2020) noted that Weather forecasting has been a tool of decision making since millennia. In connection with this Lawrence (2021a), also found that until about 350 years ago scientists and natural philosophers thought that the Sun, Moon and planets play an important role in modulating the weather on Earth.

The prediction that is set up via the observation of the moon would offer sufficient lapse time in a more than/within ahead of the three months lead time and is more accurate than the other traditional forecasting objects that are, currently, under use in the study area. In addition, this future seasonal weather indicating features of the moon would provide specific spatial dimensions of the weather events within and beyond the Borana zone area in all direction. Especially, future phenomena of the severity and physical distribution of drought; and location specific amount of rainfall in the Borana zone and its surrounding area is easily predicted by using seasonal weather indicating features of the moon. This is agreed with the finding of the Lawrence (2021b), which noted that many village level Astrologers (Pandits) are still predicting highly accurate weather with planet movements in advance of the occurrences of the events which allow people to prepare in disaster risk management.

These outlooks of the weather indicating features of the moon which are used for making seasonal weather forecasting by Borana pastoralists are categorized in to two major sets of the multidimensional distinctive interrelated patterns. These outlooks of the Moon includes: 1) weather indicating temporal patterns of the moon/patterns of the days of the emerging of the new Moon Locally called *ayyaana Ji’aa*) ^1^, 2) Weather indicating physical pattern of the moon, In line with this, a physical pattern of the Moon entails four observable indicators during the dry season of *Bona Adolessa* and *Bona Hagaya* to make the probable clue of the upcoming rainfall season: Moon with tiny synthesized cloud, Moon with partially synthesized cloud, Moon without synthesized cloud and; the Color patterns of the observable external physical quality/dirtiness of the outlooks of the moon which are changed periodically. The weather phenomena indicating temporal patterns of the moon are categorized in to two interrelated patterns: 1) Day Patterns of the Moon, and 2) rainfall patterns of the day of the new moon. Study of Oruru *et al.* (2016), explored that indigenous people in Uganda observe moon and stars to predict weather changes.

### 2.2 Weather indicating temporal patterns of the Moon

In line with this, future weather indicating temporal patterns of the moon are categorized in to two interrelated patterns: 1) Day patterns of the Moon ( Tables 1- 12), and 2) rainfall patterns of the moon.

**Table 1.** Indigenous knowledge based twenty seven days of the Moon of the year.

SN	Names of the 27 days of the Moon	Name of the Moon which is emerge on each Month of the year
1	*Gardaduma*	September ( *Birraa*)
2	*Sonsa*	
3	*Ruruma*	October ( *Ciqaawaa*)
4	*Lumasa*	
5	*Gidada*	November ( *Sadaasa*)
6	*Ruda*	
7	*Areeri duraa*	December ( *Arfaasa*)
8	*Areeri bal’aa*	
9	*Adula duraa*	January ( *Amaajji*)
10	*Adula bal’aa*	
11	*Garba duraa*	February ( *Guraandhala*)
12	*Garba bal’aa*	
13	*Garba dullacha*	
14	*Bita duraa*	March ( *Bitotessa*)
15	*Bita bal’aa*	
16	*Sorsa*	April ( *Caamsaa*)
17	*Algajim*	
18	*Arba*	May ( *Buufaa*)
19	*Walla*	
20	*Basa dura*	June ( *Wacabaji*)
21	*Basa bal’aa*	
22	*Maganata duraa (Carraa)*	July ( *obora* Guddaa)
23	*Maganata Jarraa*	
24	*Maganata biritii*	
25	*Salban duraa*	August ( *Oboraxiqqaa*)
26	*Salbaan bal’aa*	
27	*Salbaan dullacha*	

Source: Synthesized idea of Research Participant, 2021.

**Table 2.** Weather indicating temporal patterns of the January Moon.

SN	Dates of the January Moon/Emerging phase	Lunar cycles	SN	Abstract cycles/hidden phase
1	*Adula duraa*	Light month of the moon	*28*	Full dark night
2	*Adula bal’aa*		*29*	
			*30*	
3	*Garba duraa*			
4	*Garba bal’aa*			
5	*Garba dullacha*			
6	*Bita duraa*			
7	*Bita bal’aa*			
8	*Sorsa*			
9	*Algajim*			
10	*Arba*			
11	*Walla*			
12	*Basa dura*			
13	*Basa bal’aa*			
14	*Maganata duraa (Carraa)*			
15	*Maganata jarraa*			
16	*Maganata biritii*	Dark month of the moon		
17	*Salban duraa*			
18	*Salbaan bal’aa*			
19	*Salbaan dullacha*			
20	*Gardaduma*			
21	*Sonsa*			
22	*Ruruma*			
23	*Lumasa*			
24	*Gidada*			
25	*Ruda*			
26	*Areeri duraa*			
27	*Areeri bal’aa*	

Source: RP, 2021.

**Table 3.** Weather indicating temporal patterns of the February Moon.

SN	Dates of the February Moon/emerging phase	Lunar cycles	SN	Abstract cycles/hidden phase
1	*Garba duraa*	Light month of the moon	28	Full dark night
2	*Garba bal’aa*		29	
3	*Garba dullacha*		30	
			31	
4	*Bita duraa*			
5	*Bita bal’aa*			
6	*Sorsa*			
7	*Algajim*			
8	*Arba*			
9	*Walla*			
10	*Basa dura*			
11	*Basa bal’aa*			
12	*Maganata duraa*			
13	*Maganata Jarraa*			
14	*Maganata biritii*			
15	*Salban duraa*			
16	*Salbaan bal’aa*			
17	*Salbaan dullacha*	Dark month of the moon		
18	*Gardaduma*			
19	*Sonsa*			
20	*Ruruma*			
21	*Lumasa*			
22	*Gidada*			
23	*Ruda*			
24	*Areeri duraa*			
25	*Areeri bal’aa*			
26	*Adula duraa*			
27	*Adula bal’aa*	

Source: RP, 2021.

**Table 4.** Weather indicating temporal patterns of the March Moon.

SN	Dates of the March Moon	Lunar cycles	SN	Lunar abstract cycles
*1*	*Bita duraa*	Light month of the moon	*28*	Full dark night
*2*	*Bita bal’aa*		*29*	
			*30*	
*3*	*Sorsa*			
*4*	*Algajim*			
*5*	*Arba*			
*6*	*Walla*			
*7*	*Basa dura*			
*8*	*Basa bal’aa*			
*9*	*Maganata duraa (Carraa)*			
*10*	*Maganata Jarraa*			
*11*	*Maganata biritii*			
*12*	*Salban duraa*			
*13*	*Salbaan bal’aa*			
*14*	*Salbaan dullacha*			
*15*	*Gardaduma*			
*16*	*Sonsa*	Dark month of the moon		
*17*	*Ruruma*			
*18*	*Lumasa*			
*19*	*Gidada*			
*20*	*Ruda*			
*21*	*Areeri duraa*			
*22*	*Areeri bal’aa*			
*23*	*Adula duraa*			
*24*	*Adula bal’aa*			
*25*	*Garba duraa*			
*26*	*Garba bal’aa*			
*27*	*Garba dullacha*	

Source: RP, 2021.

**Table 5.** Weather indicating temporal patterns of the April Moon.

SN	Days of the April Moon/emerging phase	Lunar cycles	SN	Abstract cycles/hidden phase
1	*Sorsa*	Light month of the moon	28	Full dark night
2	*Algajim*		29	
			30	
3	*Arba*			
4	*Walla*			
5	*Basa dura*			
6	*Basa bal’aa*			
7	*Maganata duraa (Carraa)*			
8	*Maganata Jarraa*			
9	*Maganata biritii*			
10	*Salban duraa*			
11	*Salbaan bal’aa*			
12	*Salbaan dullacha*			
13	*Gardaduma*			
14	*Sonsa*			
15	*Ruruma*			
16	*Lumasa*	Dark month of the moon		
17	*Gidada*			
18	*Ruda*			
19	*Areeri duraa*			
20	*Areeri bal’aa*			
21	*Adula duraa*			
22	*Adula bal’aa*			
23	*Garba duraa*			
24	*Garba bal’aa*			
25	*Garba dullacha*			
26	*Bita duraa*			
27	*Bita bal’aa*	

Source: RP, 2021.

**Table 6.** Weather indicating temporal patterns of the May Moon.

SN	Dates of the May Moon/Emerging phase	Lunar cycles	SN	Abstract cycles/hidden phase
1	*Arba*	Light month of the moon	28	Full dark night
2	*Walla*		29	
			30	
3	*Basa dura*			
4	*Basa bal’aa*			
5	*Maganata duraa (Carraa)*			
6	*Maganata Jarraa*			
7	*Maganata biritii*			
8	*Salban duraa*			
9	*Salbaan bal’aa*			
10	*Salbaan dullacha*			
11	*Gardaduma*			
12	*Sonsa*			
13	*Ruruma*			
14	*Lumasa*			
15	*Gidada*			
16	*Ruda*	Dark month of the moon		
17	*Areeri duraa*			
18	*Areeri bal’aa*			
19	*Adula duraa*			
20	*Adula bal’aa*			
21	*Garba duraa*			
22	*Garba bal’aa*			
23	*Garba dullacha*			
24	*Bita duraa*			
25	*Bita bal’aa*			
26	*Sorsa*			
27	*Algajim*	

Source: RP, 2021.

**Table 7.** Weather indicating temporal patterns of the June Moon.

SN	Days of the June Moon/emerging phase	Lunar cycles	SN	Abstract cycles/hidden phase
1	*Basa dura*	Light month of the moon	28	Full dark night
2	*Basa bal’aa*		29	
			30	
3	*Maganata duraa (Carraa)*			
4	*Maganata Jarraa*			
5	*Maganata biritii*			
6	*Salban duraa*			
7	*Salbaan bal’aa*			
8	*Salbaan dullacha*			
9	*Gardaduma*			
10	*Sonsa*			
11	*Ruruma*			
12	*Lumasa*			
13	*Gidada*			
14	*Ruda*			
15	*Areeri duraa*			
16	*Areeri bal’aa*	Dark night month of the moon		
17	*Adula duraa*			
18	*Adula bal’aa*			
19	*Garba duraa*			
20	*Garba bal’aa*			
21	*Garba dullacha*			
22	*Bita duraa*			
23	*Bita bal’aa*			
24	*Sorsa*			
25	*Algajim*			
26	*Arba*			
27	*Walla*	

Source: RP, 2021.

**Table 8.** Weather indicating temporal patterns of the July Moon.

SN	Dates of the July Moon/emerging phase	Lunar cycles	SN	Abstract cycles/hidden phase
1	*Maganata duraa (Carraa)*	Light month of the moon	28	Full dark night
2	*Maganata Jarraa*		29	
3	*Maganata biritii*		30	
			31	
4	*Salban duraa*			
5	*Salbaan bal’aa*			
6	*Salbaan dullacha*			
7	*Gardaduma*			
8	*Sonsa*			
9	*Ruruma*			
10	*Lumasa*			
11	*Gidada*			
12	*Ruda*			
13	*Areeri duraa*			
14	*Areeri bal’aa*			
15	*Adula duraa*			
16	*Adula bal’aa*	Dark night month of the moon		
17	*Garba duraa*			
18	*Garba bal’aa*			
19	*Garba dullacha*			
20	*Bita duraa*			
21	*Bita bal’aa*			
22	*Sorsa*			
23	*Algajim*			
24	*Arba*			
25	*Walla*			
26	*Basa dura*			
27	*Basa bal’aa*	

Source: RP, 2021.

**Table 9.** Weather indicating temporal patterns of the August Moon.

SN	Dates of the August Moon/emerging phase	Lunar cycles	SN	Abstract cycles/hiddenphase
1	*Salban duraa*	Light month of the moon	28	Full dark night
2	*Salbaan bal’aa*		29	
3	*Salbaan dullacha*		30	
			31	
4	*Gardaduma*			
5	*Sonsa*			
6	*Ruruma*			
7	*Lumasa*			
8	*Gidada*			
9	*Ruda*			
10	*Areeri duraa*			
11	*Areeri bal’aa*			
12	*Adula duraa*			
13	*Adula bal’aa*			
14	*Garba duraa*			
15	*Garba bal’aa*			
16	*Garba dullacha*	Dark month of the moon		
17	*Bita duraa*			
18	*Bita bal’aa*			
19	*Sorsa*			
20	*Algajim*			
21	*Arba*			
22	*Walla*			
23	*Basa dura*			
24	*Basa bal’aa*			
25	*Maganata duraa (Carraa)*			
26	*Maganata Jarraa*			
27	*Maganata biritii*			

Source: Synthesized idea of RP, 2021.

**Table 10.** Weather indicating temporal patterns of the September Moon.

SN	Days of the September Moon/emerging phase	Lunar cycles	SN	Abstract cycles/hidden phase
1	*Gardaduma*	Light month of the moon	*28*	Full dark night
2	*Sonsa*		*29*	
			*30*	
3	*Ruruma*			
4	*Lumasa*			
5	*Gidada*			
6	*Ruda*			
7	*Areeri duraa*			
8	*Areeri bal’aa*			
9	*Adula duraa*			
10	*Adula bal’aa*			
11	*Garba duraa*			
12	*Garba bal’aa*			
13	*Garba dullacha*			
14	*Bita duraa*			
15	*Bita bal’aa*			
16	*Sorsa*	Dark night month of the moon		
17	*Algajim*			
18	*Arba*			
19	*Walla*			
20	*Basa dura*			
21	*Basa bal’aa*			
22	*Maganata duraa (Carraa)*			
23	*Maganata Jarraa*			
24	*Maganata biritii*			
25	*Salban duraa*			
26	*Salbaan bal’aa*			
27	*Salbaan dullacha*	

Source: RP, 2021.

**Table 11.** Weather indicating temporal patterns of the October Moon.

SN	Days of the October Moon/emerging phase	Lunar cycles	SN	Abstract cycles/hidden phase
1	*Ruruma*	Light month of the moon	28	Full dark night
2	*Lumasa*		29	
			30	
3	*Gidada*			
4	*Ruda*			
5	*Areeri duraa*			
6	*Areeri bal’aa*			
7	*Adula duraa*			
8	*Adula bal’aa*			
9	*Garba duraa*			
10	*Garba bal’aa*			
11	*Garba dullacha*			
12	*Bita duraa*			
13	*Bita bal’aa*			
14	*Sorsa*			
15	*Algajim*			
16	*Arba*	Dark month of the moon		
17	*Walla*			
18	*Basa dura*			
19	*Basa bal’aa*			
20	*Maganata duraa (Carraa)*			
21	*Maganata Jarraa*			
22	*Maganata biritii*			
23	*Salban duraa*			
24	*Salbaan bal’aa*			
25	*Salbaan dullacha*			
26	*Gardaduma*			
27	*Sonsa*	

Source: RP, 2021.

**Table 12.** Weather indicating temporal patterns of the November Moon.

SN	Dates of the November Moon/emerging phase	Lunar cycles	SN	Abstract cycles/hidden phase
1	*Gidada*	Light month of the moon	28	Full dark night
2	*Ruda*		29	
			30	
3	*Areeri duraa*			
4	*Areeri bal’aa*			
5	*Adula duraa*			
6	*Adula bal’aa*			
7	*Garba duraa*			
8	*Garba bal’aa*			
9	*Garba dullacha*			
10	*Bita duraa*			
11	*Bita bal’aa*			
12	*Sorsa*			
13	*Algajim*			
14	*Arba*			
15	*Walla*			
16	*Basa dura*	Dark month of the moon		
17	*Basa bal’aa*			
18	*Maganata duraa (Carraa)*			
19	*Maganata Jarraa*			
20	*Maganata biritii*			
21	*Salban duraa*			
22	*Salbaan bal’aa*			
23	*Salbaan dullacha*			
24	*Gardaduma*			
25	*Sonsa*			
26	*Ruruma*			
27	*Lumasa*	

Source: RP, 2021.

**Day patterns of the Moon**: According to local calendar there are abstractly made 27 distinctive day patterns of a moon which are locally called *Ayyaana.* The main legends of the temporal patterns of the moon days are to ratify the days on which each new moon are emerging/re-emerging in the night sky. In the study area; each moon has distinctive temporal patterns. This study discover that Borana Oromo abstractly created astronomic based sequential list of 27 traditional temporal patterns of the moon is reconfigured/varied in chronological order with in each of the 12 moon/months of the year. This day/temporal patterns of a new moon have great influence on the upcoming season weather phenomena and livelihood decision making process. This is in consistent with the, Baki study in Botswana quoted in (Loretta, 2014) posited that celestial bodies are used for practical purposes such as timekeeping and in creating accurate calendar; is therefore fundamental in relation to the daily cycle of work as well as the annual round of agricultural activities. In similar with this, study in ancient weather forecasting de-signs ( Beardmore, 2013) noted that astronomy, Weather and Calendars are the main methods of weather prediction in the ancient world.

Table 1 discloses sequential lists of the days of a month which are the benchmark/point of a reference for the description of the next 12 Tables. Generation old knowledge of Borana Oromo abstractly created sequential list of 27 traditional day patterns that are derived/developed/based on the lunar cycles of the light and dark nights of the moon in the sky are depicted in the Table 1. These 27 days of a month that are traditionally derived from the lunar cycles of the moon which are reconfigured/varied in chronological order with in each of the 12 moons of the year are used for weather forecasting since time immemorial, in the study area (see all 12 tables).

**Remark1, First day of the new moon**: As depicted in the Table 1, in the study area; the start/commence of the first day of each moon (in the column 3 of Table 1) begin from the first probable day/as listed in the column 2 of Table 1) on which each particular new moon is expected to re-emerge in the night sky. Out of 12 moons of the year; nine of them have nature driven two probable emerging days as listed in the column 2 of Table 1(which makes them 30 days per month) and three of them have three probable emerging days (which makes them 31 days per month). In this regard, in the study area; for the purpose of annual/monthly calendar commencements of the first date of the new moon are begin from the first probable day of their re-observation in the night sky. However, for the purpose of the seasonal calendar; commencements of the first date of the new moon are beginning from the first actual emerging day of their re-observation in the night sky. Study area community believes that the exact actual first date of the emergence of the new moon has great impact on the upcoming season weather phenomena.

**Remark 2, Full Dark Night of the Moon/End Date of each Moon:** each moon of a year has one full dark night day. In this regard, there is no observable sign of moon in the night sky on full dark night sky day. This full dark night day is locally called *Luwoo* which is between the *Diba* of the current moon and the crescent of the next new moon. Traditionally, this day is expected as the driver of the terrifying events. Hence, it is not listed with in the nature driven locally created abstract sequence of the 27 dates of a moon; and it is silently over passed by the community in the study area. It is the end date of each moon; it is the 30 ^th^ date for those of the moon having two probable emerging day; and 31 ^st^ date for those of the moon having three probable emerging day.

**Remark 3, Double Count Dates**: As stated in the remark one and two of this section; each 12 moon of a year has two/three probable emerging days. Local community believes that each moon of a year has emerging phase (1 ^st^ to 15 ^th^ dates) and hidden phase (16 ^th^ to 30/31 ^th^ dates) as depicted in the Tables 1– 12. In this case, they believed that the first date of the emerging phase of each moon in the column 3 of Table 1 begin from the first probable emerging date that are listed orderly in the column two of Table 1. Similarly, the 30 ^th^/31 ^st^ date of the final hidden phase of each moon is/was completed on the *Luwoo* day of each moon that was represented by the serial number 30/31 in the column 4 of the Tables 1– 12 in this section. This implies that, 1 ^st^, 2 ^nd^ and 3 ^rd^ dates are re-counted/double counted in the IK based calendar of the study area. Hence, this study constructed two columns of serial number in all 1–12 Tables that was derived from the benchmark Table 1, in this section. The serial numbers 1 to 27 were listed in the first column of the Tables to orderly count 1 ^st^ to 27 ^th^ dates in the emerging phase of the moon. The serial number 28, 29, 30/31were listed in the column 4 of all 1–12 Tables to re-count/double count hidden phase of the moon. This was due to the fact that the two/three probable emerging dates (represented by serial number 1 to 3) of the new moon in the column 2 of 1–12 Tables were recounted in the hidden phase of the dates (represented by the serial number 28 to 30/31) of the end of each moon.

As depicted here below; all the 12 Tables in this section/seasonal weather indicating temporal patterns of the moon/were derived from the Table 1.

As it is seen from Table 2, January moon is emerged on the day *of Adula duraa.* Lunar cycle of 1 ^st^ up to 15 ^th^dates of the January moon ( *Adula duraa up to Maganata jarraa)* are locally called the light/bright moon nights of the January Month ^2^. Lunar cycle of the 16 ^th^ up to 30 ^th^ dates of the January moon ( *Maganata biritii* up to *Adula bal’aa*) are locally called the dark moon nights of the January Month. When it is seen, literally January moon has only 29 days. However, it had 30 days. In this case, next to the *day* of *Adula bal’aa/next to serial number 29* in the hidden phase of the double counting/ there was one full dark night without no sign of moon in the night sky which is locally called *Luwoo* of January moon/30 ^st^ date of January month. In line with this, because of the January Moon has two probable emerging days which consists of *Adula duraa* and *Adula bal’aa*/serial number 1 to 2 in the emerging phase of the moon/the total dates of the January moon are/were 30. The *Diba* (final hardly observable decreased margin in the hidden phase of the moon) of the January moon was observed on the date of the *Adula bal’aa* which was represented by the serial number 29 of the column 4 in the Table 2. The probability of the emerging of the January moon is on the day of *Adula duraa (serial number 1) or Adula bal’aa (serial number 2).* In this case, there is a common belief that when the January moon is emerged on the day of *Adula duraa; the upcoming Ganna* rain season (March to May) would be very little/the probability of severe drought would be very high. In the 2021, January moon was emerged on the day of *Adula bal’aa* instead of *Adulaa duraa.* Hence, the study community believed that severe and devastating drought of 2021/2022 was mainly caused by the nature driven anomalous of the emerging of the January Moon of 2021 on the day of *Adulaa duraa.*

This is in agreement with the study of Lee *et al.* (2020) which noted that, the regularity of the motions of celestial objects enabled peoples around the world to create worldviews that is culturally organized systems of knowledge and generations of sky watchers, carefully tracking the positions of the heavenly bodies in order to understand how to conduct the human life on the earth. In addition, study quoted in Loretta (2014) noted that the perspective of the Mapuche people of Chile belief as natural forces govern and regulate the universe. It also stated that there is a very strong connection with nature and the environment in which one lives.

As it is seen from Table 3, February moon is emerged on the day *of Garba Duraa.* Lunar cycle of 1 ^st^ up to 15 ^th^ dates of the February moon ( *Garba Duraa up to Salban Duraa*) are locally called the light/bright moon nights of the February Month ^3^. Lunar cycle of the 16 ^th^ up to 31 ^st^ dates of the February moon ( *SalbanBall’aa* up to *Garba Dullacha*) are locally called the dark moon nights of the February Month. When it is seen, literally February moon has only 30 days. However, it has 31 days. In this case, next to the *day* of *Garba dullacha/next to serial number 30* in the hidden phase of the double counting within the column 4 of the Table 3/ there was one full dark night without no sign of moon in the night sky which is locally called *Luwoo* of February moon/31 ^st^ date of February month. In line with this, because of the February Moon has three probable emerging days which consists of *Garba Duraa, Garba Bal’aa* and *Garba Dullacha*/serial number 1 to 3 in the emerging phase of the moon/the total dates of the February moon are/were 31. The *Diba* (final hardly observable decreased margin in the hidden phase of the moon) of the February moon was on the day of the *Garba Dullachaa which was represented by the serial number 30 of the column 4 in the* *Table 3.* Finally, there was one full dark night on which there was completely no moon which is locally called *Luwoo* of the February moon or the 31 ^st^ day of the February month. The probability of the emerging of the February moon is on the day of *Garba Duraa, Garba Bal’aa* and *Garba Dullacha.* In this case, when the February moon is emerged on the day of *Garba Duraa* the upcoming Ganna rainfall (March to May) would be very little/the probability of prolonged and severe drought in the *Bona addolessa* (December to February) would be very high. In the 2021, February moon was emerged on the day of *Garba duraa instead of Garba Bal’aa.* Furthermore, community believed that when the shower of rainfall is occurred on either days of *Gardaduma, Sonsa, Ruruma, Lumasa, Gidada, Arba, Walla, Sorsa, Algajim and Ruda* of the February month; the upcoming *Ganna* rainfall (March to May) would be very little. In this case, shower of rainfall was occurred on the day of *Sonsa in February* 2021. Hence, the study community believed that severe and devastating drought of 2021/2022 was mainly caused by the nature driven anomalous of the emerging of the February Moon of 2021 on the day of *Garba duraa* and occurrence of shower of rainfall on the day of *Sonsa.*

As it is seen from Table 4, March moon is emerged on the day *of Bita duraa.* Lunar cycle of 1 ^st^ up to 15 ^th^ dates of the March moon ( *Bita duraa up to Gardaduma)* are locally called the light/bright moon nights of the March Month ^4^. Lunar cycle of the 16 ^th^ up to 30 ^th^ dates of the March moon ( *Sonsa* up to *Bita bal’aa*) are locally called the dark moon nights of the March Month. When it is seen, literally March moon has only 29 days. However, it had 30 days. In this case, next to the *day* of *Bita bal’aa/next to serial number 29* in the hidden phase of the double counting/ there was one full dark night without no sign of moon in the night sky which is locally called *Luwoo* of March moon/30 ^th^ date of March month. In line with this, because of the March Moon have two probable emerging days which consists of *Bita duraa* and *Bita bal’aa*/serial number 1 to 2 in the emerging phase of the moon/the total days of the March moon are/were 30. The *Diba* (final hardly observable decreased margin in the hidden phase of the moon) of the March moon was observed on the day of the *Bita bal’aa which was represented by the serial number 29 of the column 4 inthe* *Table 4.* The probability of the emerging of the March moon is on the day of *Bita duraa (serial number 1) or Bita bal’aa (serial number 2).* In this case, there is a common belief that when the March moon is emerged on the day of *Bita duraa; upcoming Ganna* rain season (March to May) would be very little/the probability of prolonged and severe drought would be very high in the *Bona Hagayyaa (dry season of December to February).* In the 2021, March moon was emerged on the day of *Bita duraa instead of Bita bal’aa.* Furthermore, community believed that when the first shower rainfall is occurred on either days of *Gardaduma, Sonsa, Ruruma, Lumasa, Gidada, Arba, Walla, Sorsa, Algajim and Ruda* of the March month; the *Ganna* rainfall (March to May) would be very little in its entire dimension. In this case, first shower of 2021 *Ganna* rainfall (March to May) was occurred on the day of *Gardaduma.* Hence, the study community believed that severe and devastating drought of 2021/2022 was mainly caused by the nature driven anomalous of the emerging of the March Moon of 2021 on the day of *Bita duraa*; and the occurrence of the first shower of 2021 Ganna rainfall (March to May) on the dates of *Gardaduma.*

This is in agreed with the study of Scofield (2010) in the ancient Greece weather forecasting de-signs. Hesiod used three counting traditions to describe the various points in the Moon's cycle that are favorable or unfavorable for one activity or another. He first counted the entire cycle, referring to the positive qualities of the first, fourth, and seventh days of the Moon for planting. The first day is being the day on which the crescent appears after the New Moon; about 1 to 15 days after the New Moon. He then shifted into another way of counting the cycle; stating that the sixteenth day of the mid-month is bad for plants. The second cycle is also the 16th day of the Moon/the day after the Full Moon. Study of Beardmore (2013) also supported this study which stated that Hesiod's good and bad days of the lunar month, appears to be a kind of indigenous Greek astrology that offered information for farmers. This justification illustrated as moon days are used for livelihood decision making in the ancient Greece, however, it is yet not stated the future temporal, spatial and features of the future/seasonal/weather indicating outlooks of the moon.

As it is seen from Table 5, April moon is emerged on the day *of Sorsa.* Lunar cycle of 1 ^st^ up to 15 ^th^ dates of the March moon ( *Sorsa up to Ruruma)* are locally called the light/bright moon nights of the April Month ^5^. Lunar cycle of the 16 ^th^ up to 30 ^th^ dates of the April moon ( *Lumasa* up to *Algajim*) are locally called the dark moon nights of the April Month. When it is seen, literally April moon has only 29 days. However, it had 30 days. In this case, next to the *day* of *Algajim/next to serial number 29* in the hidden phase of the double counting/ there was one full dark night without no sign of moon in the night sky which is locally called *Luwoo* of April moon/30 ^th^ date of April month. In line with this, because of the April Moon has two probable emerging days which consists of *Sorsa* and *Algajim*/serial number 1 to 2 in the emerging phase of the moon/the total days of the April moon are/were 30. The *Diba* (final hardly observable decreased margin in the hidden phase of the moon) of the April moon was observed on the day of the *Algajim which was represented by the serial number 29 of the column 4 in the* *Table 5.* The probability of the emerging of the April moon is on the days of *Sorsa (serial number 1) or Algajim (serial number 2).* In this case, there is a common belief that when the April moon is emerged on the day of *Sorsa; Ganna* rain season (March to May) would be interupted/the probability of severe drought would be very high. In the 2021, April moon was emerged on the day of *Sorsa instead of Algajim.* Hence, the study community believed that severe and devastating drought of 2021/2022 was mainly caused by the nature driven anomalous of the emerging of the April Moon of 2021 on the day of *Sorsa*

As it is seen from Table 6, May moon is emerged on the day *of Arba.* Lunar cycle of 1 ^st^ up to 15 ^th^ dates of the May moon ( *Arba up to Gidada)* are locally called the light/bright moon nights of the May Month ^6^. Lunar cycle of the 16 ^th^ up to 30 ^th^ dates of the May moon ( *Ruda* up to *Walla*) are locally called the dark moon nights of the May Month. When it is seen, literally May moon has only 29 days. However, it had 30 days. In this case, next to the *day* of *Walla/next to serial number 29* in the hidden phase of the double counting/ there was one full dark night without no sign of moon in the night sky which is locally called *Luwoo* of May moon/30 ^th^ date of May month. In line with this, because of the May Moon has two probable emerging days which consists of *Arba* and *Walla*/serial number 1 to 2 in the emerging phase of the moon/the total days of the May moon are/were 30. The *Diba* (final hardly observable decreased margin in the hidden phase of the moon) of the May moon was observed on the day of the *Walla which was represented by the serial number 29 of the column 4 in the* *Table 6.* The probability of the emerging of the May moon is on the dates of *Arba (serial number 1) or Walla (serial number 2).* In this case, there is a common belief that when the May moon is emerged on the day of *Sorsa; Ganna* rain season (March to May) would be interrupted/the probability of severe drought would be very high. Even though, May moon of the 2021was emerged on its right day on Walla; *Ganna* rainfall (March to May) was very poor.

As it is seen from Table 7, June moon is emerged on the Day *of Basa dura.* Lunar cycle of 1 ^st^ up to 15 ^th^ dates of the June moon ( *Basa dura up to Areeri duraa)* are locally called the light/bright moon nights of the June Month Lunar cycle of the 16 ^th^ up to 30 ^th^ dates of the June moon ( *Areeri bal’aa* up to *Basa bal’aa*) are locally called the dark moon nights of the June Month. When it is seen, literally June moon has only 29 days. However, it had 30 days. In this case, next to the *day* of *Basa bal’aa/next to serial number 29* in the hidden phase of the double counting/ there was one full dark night without no sign of moon in the night sky which is locally called *Luwoo* of June moon/30 ^st^ date of June month. In line with this, because of the June Moon has two probable emerging days which consists of *Basa dura* and *Basa bal’aa*/serial number 1 to 2 in the emerging phase of the moon/the total days of the June moon are/were 30. The *Diba* (final hardly observable decreased margin in the hidden phase of the moon) of the June moon was observed on the day of the *Basa bal’aa which was represented by the serial number 29 of the column 4 in the* *Table 7.* The probability of the emerging of the June moon is on the day of *Basa dura (serial number 1) or Basa bal’aa (serial number 2).* In this case, there is a common belief that when the June moon is emerged on the day of *Basa dura; the upcoming Hagayyaa* rain season (September to October) would be very little/the probability of severe drought would be very high. In the 2021, June moon was emerged on the day of *Basa dura instead of Basa bal’aa.* Hence, the study community believed that lack of Hagayya rain/severe and devastating drought of 2021/2022 were mainly caused by the nature driven anomalous of the emerging of the June Moon of 2021 on the day of *Basa dura.*

As it is seen from Table 8, July moon is emerged on the day *of Maganata duraa.* Lunar cycle of 1 ^st^ up to 15 ^th^ day of the July moon ( *Maganata duraa up to Adula duraa)* are locally called the light/bright moon nights of the July Month Lunar cycle of the 16 ^th^ up to 31 ^st^ dates of the July moon ( *Adula bal’aa* up to *Maganata biritii*) are locally called the dark moon nights of the July Month. When it is seen, literally July moon has only 30 days. However, it had 31 days. In this case, next to the *day* of *Maganata biritii/next to serial number 30* in the hidden phase of the double counting/ there was one full dark night without no sign of moon in the night sky which is locally called *Luwoo* of July moon/31 ^st^ date of July month. In line with this, because of the July Moon has three probable emerging days which consists of *Maganata duraa, Maganata Jarraa* and *Maganata biritii*/serial number 1 to 3 in the emerging phase of the moon/the total days of the July moon are/were 31. The *Diba* (final hardly observable decreased margin in the hidden phase of the moon) of the July moon was observed on the day of the *Maganata biritii which was represented by the serial number 30 of the column 4 in the* *Table 8.* The probability of the emerging of the July moon is on the day of *Maganata duraa (serial number 1) or Maganata Jarraa (serial number 2) or Maganata biritii.* In this case, there is a common belief that when the July moon is emerged on the day of *Maganata duraa; the upcoming Hagayyaa* rain season (September to October) would be very little/the probability of severe drought in the *Bona Hagayyaa* (December to February) would be very high. In the 2021, July moon was emerged on the day of *Maganata duraa instead of Maganata Jarraa.* Hence, the study community believed that severe and devastating drought of 2021/2022 was mainly caused by the nature driven anomalous of the emerging of the July Moon of 2021 on the day of *Maganata duraa.*

As it is seen from Table 9, August moon is emerged on the day of *Salban duraa.* Lunar cycle of 1 ^st^ up to 15 ^th^ dates of the August moon ( *Salban duraa* up to *Garba bal’aa*) are locally called the light/bright moon nights of the August Month Lunar cycle of the 16 ^th^ up to 31 ^st^ dates of the August moon ( *Garba dullacha* up to *Salbaan dullacha*) are locally called the dark moon nights of the August Month. When it is seen, literally August moon has only 30 days. However, it had 31 dates. In this case, next to the day of *Salbaan dullacha* /next to serial number 30 in the hidden phase of the double counting/ there was one full dark night without no sign of moon in the night sky which is locally called *Luwoo* of August moon/31 ^st^ date of August month. In line with this, because of the August Moon has three probable emerging days which consists of *Salban duraaSalbaan bal’aa,* and *Salbaan dullacha*/serial number 1 to 3 in the emerging phase of the moon with in the column 2/the total days of the August moon are/were 31. The *Diba* (final hardly observable decreased margin in the hidden phase of the moon) of the August moon was observed on the day of the *Salbaan dullacha* which was represented by the serial number 30 of the column 4 in the Table 9. The probability of the emerging of the August moon is on the day of *Salban duraa* (serial number 1) or *Salbaan bal’aa* (serial number 2) or *Salbaandullacha (serial number 3).* In this case, there is a common belief that when the August moon is emerged on the day of *Salbanduraa*; the upcoming *Hagayyaa* rainfall season (September to October) would be very little/the probability of severe drought would be very high. In the 2021, August moon was emerged on the day of *Salban duraa* instead of *Salbaan bal’aa.* Hence, the study community believed that severe and devastating drought of 2021/2022 was mainly caused by the nature driven anomalous of the emerging of the August Moon of 2021 on the dates of *Salban duraa.* This is supported by the Study in India ( Sankar *et al.* 2019) posited that Many village level Astrologers (Pandits) are still predicting highly accurate weather with planet movements. It also stated that the daily rainfall forecast through Astro-meteorology showed the highest forecast accuracy of 74–87 percent. Apparently study undertaken by Lawrence (2021b) explore that the repeated cycles of the sun, moon, and stars helped to regulate human activity as people strove to make sense of their world and to keep their actions in harmony with the cosmos as they perceived it. Study of ( Scofield, 2010) found that early Greece use context of astrological features to best illustrate works and days, the cycle of the year, and the month in the process of agricultural activities.

As it is seen from Table 10, September moon is emerged on the day *of Gardaduma.* Lunar cycle of 1 ^st^ up to 15 ^th^ dates of the September moon ( *Gardaduma to Bita bal’aa)* are locally called the light/bright moon nights of the September Month Lunar cycle of the 16 ^th^ up to 30 ^th^ dates of the September moon ( *Sorsa* up to *Sorsa*) are locally called the dark moon nights of the September Month. When it is seen, literally September moon has only 29 days. However, it had 30 days. In this case, next to the *day* of *Sonsa /next to serial number 29* in the hidden phase of the double counting/ there was one full dark night without no sign of moon in the night sky which is locally called *Luwoo* of September moon/30 ^th^ date of September month. In line with this, because of the September Moon has two probable emerging dates which consists of *Gardaduma* and *Sonsa*/serial number 1 to 2 in the emerging phase of the moon/the total days of the September moon are/were 30. The *Diba* (final hardly observable decreased margin in the hidden phase of the moon) of the September moon was observed on the day of the *Sonsa which was represented by the serial number 29 of the column 4 in the* *Table 10.* The probability of the emerging of the September moon is on the day of *Gardaduma (serial number 1) or Sonsa (serial number 2).* In this case, there is a common belief that when the September moon is emerged on the day the upcoming of *Gardaduma; Hagayyaa* rain season (September to October) would be very little/the probability of severe drought would be very high.

As it is seen from Table 11, October moon is emerged on the day *of Ruruma.* Lunar cycle of 1 ^st^ up to 15 ^th^ dates of the October moon ( *Ruruma up to Algajim)* are locally called the light/bright moon nights of the October Month ^7^. Lunar cycle of the 16 ^th^ up to 30 ^th^ dates of the October moon ( *Arba* up to *Lumasa*) are locally called the dark moon nights of the October Month. When it is seen, literally October moon has only 29 days. However, it had 30 days. In this case, next to the *day* of *Lumasa/next to serial number 29* in the hidden phase of the double counting/ there was one full dark night without no sign of moon in the night sky which is locally called *Luwoo* of October moon/30 ^th^ date of October month. In line with this, because of the October Moon has two probable emerging days which consists of *Ruruma* and *Lumasa/*serial number 1 to 2 in the emerging phase of the moon/the total days of the October moon are/were 30. The *Diba* (final hardly observable decreased margin in the hidden phase of the moon) of the October moon was observed on the day of the *Lumasa which was represented by the serial number 29 of the column 4 in the* *Table 11.* The probability of the emerging of the October moon is on the day of *Ruruma (serial number 1) or Lumasa (serial number 2).* In this case, there is a common belief that when the October moon is emerged on the day of *Ruruma; Hagaya* rain season (October to November) would be interrupted/the probability of severe drought in the Bona Hagayyaa (December to February) would be very high. In the 2021, October moon was emerged on the day of *Ruruma instead of Lumasa.* Furthermore, community believed that when the first shower of rainfall is occurred on either day of *Gardaduma, Sonsa, Ruruma, Lumasa, Gidada, Arba, Walla, Sorsa, Algajim and Ruda* of the October month; the *Hagayyaa* rainfall would be very little/interrupted early. Similarly, first shower of 2021 *October* rainfall was occurred on the day of *Ruruma.* Hence, the study community believed that severe and devastating drought of 2021/2022 was mainly caused by the nature driven anomalous of the emerging of the October Moon of 2021 on the day of *Ruruma*; and the occurrence of the first shower of 2021 *Hagayya* rainfall on the day of *Arba.* Study of Allen quoted in (Loretta, 2014) posited that indigenous peoples observations of the celestial objects give future clue of the seasonal patterns. This is exactly still functional among the Borana Oromo of Southern Ethiopia.

As it is seen from Table 12, November moon is emerged on the day *of Gidada.* Lunar cycle of 1 ^st^ up to 15 ^th^ dates of the November moon ( *Gidada up to Walla)* are locally called the light/bright moon nights of the November Month ^8^. Lunar cycle of the 16 ^th^ up to 30 ^th^ dates of the November moon ( *Basa dura* up to *Ruda*) are locally called the dark moon nights of the November Month. When it is seen, literally November moon has only 29 days. However, it had 30 days. In this case, next to the *day* of *Ruda /next to serial number 29* in the hidden phase of the double counting/ there was one full dark night without no sign of moon in the night sky which is locally called *Luwoo* of November moon/30 ^th^ date of November month. In line with this, because of the November Moon has two probable emerging days which consists of *Gidada* and *Ruda/*serial number 1 to 2 in the emerging phase of the moon/the total days of the November moon are/were 30. The *Diba* (final hardly observable decreased margin in the hidden phase of the moon) of the November moon was observed on the day of the *Ruda which was represented by the serial number 29 of the column 4 in the* *Table 12.* The probability of the emerging of the October moon is on the day of *Gidada (serial number 1) or Ruda (serial number 2).* In this case, there is a common belief that when the November moon is emerged on the day of *Gidada* the probability of prolonged/severe drought in the *Bona Hagayyaa* (December to February) and the scarcity/interruption of rainfall in the upcoming Ganna season (March to May) would be very high in the study area. Hence, the study community believed that the devastating drought of the 2021/202 drought was caused by the anomalous of the emergence of the November moon on the day of *Gidada.* Similarly ( Lehoux, 2021) found that in ancient Greek new and full moons were used to determine general weather conditions. Study of Mcmillan quoted in (Lorettaa, 2014) revealed that Batswana communities have prominent phonological markers of cultural astronomy that signal the change of the seasons; predict droughts as well as weather related diseases by watching the movements of celestial bodies.

As it is seen from Table 13, December moon is emerged on the day *of Areeri duraa.* Lunar cycle of 1 ^st^ up to 15 ^th^ dates of the December moon ( *Areeri duraa up to Basa bal’aa)* are locally called the light/bright moon nights of the December Month ^9^. Lunar cycle of the 16 ^th^ up to 30 ^th^ dates of the December moon ( *Maganata duraa* up to *Areeri bal’aa*) are locally called the dark moon nights of the December Month. When it is seen, literally December moon has only 29 days. However, it had 30 days. In this case, next to the *days* of *Areeri bal’aa /next to serial number 29* in the hidden phase of the double counting/ there was one full dark night without no sign of moon in the night sky which is locally called *Luwoo* of December moon/30 ^th^ days of December month. In line with this, because of the December Moon has two probable emerging days which consists of *Areeri duraa* and *Areeri bal’aa/*serial number 1 to 2 in the emerging phase of the moon/the total days of the December moon are/were 30. The *Diba* (final hardly observable decreased margin in the hidden phase of the moon) of the December moon was observed on the day of the *Areeri bal’aa which was represented by the serial number 29 of the column 4 in the* *Table 13.* The probability of the emerging of the October moon is on the day of *Areeri duraa (serial number 1) or Areeri bal’aa (serial number 2).* In this case, there is a common belief that when the December moon is emerged on the day of *Areeri duraa* the probability of prolonged and severe drought would be very high in the *Hagayya* drought season (December to February) and scarcity of rainfall in the upcoming Ganna season (March to May). Hence, the study community believed that the devastating drought of the 2021/202 drought was mainly caused by the anomaly of the emerging of the December moon on the day of *Areeri duraa* Rainfall Patterns of the Day of New Moon: In the study area it is believed that day of a new moon has decisive factors on the amount and distribution of rainfall. In this case, when the first shower of rainfall is occurred on the day of *Salbaan duraa;* high rainfall will be expected in that whole season (see Tables 1–12). Similarly, when the first shower of rainfall is occurred on the day of *Salban bal’aa, salban dullacha, Basa bal’aa, Areeri dura, Areeri bal’aa and Bitaa bal’aa*; sufficient rainfall is expected in that whole season. Similarly, when the first shower is occurred on the day of *Basaa duraa;* there is no equal physical distribution of rainfall in that particular season for all area of Borana zone. Similarly, when the first shower is occurred on the days of *sorsa, algajim, arba, walla, gardaduma, sonsa, ruruma, lumasa, gidada* and ruda; rainfall is not sufficient in amount and physical distributions, in the study area.

**Table 13.** Weather indicating temporal patterns of the December Moon.

SN	Days of the December Moon/emerging phase	Lunar cycles	SN	Hidden phase/abstract cycle
1	*Areeri duraa*	Light month of the moon	28	Full dark night
2	*Areeri bal’aa*		29	
			30	
3	*Adula duraa*			
4	*Adula bal’aa*			
5	*Garba duraa*			
6	*Garba bal’aa*			
7	*Garba dullacha*			
8	*Bita duraa*			
9	*Bita bal’aa*			
10	*Sorsa*			
11	*Algajim*			
12	*Arba*			
13	*Walla*			
14	*Basa dura*			
15	*Basa bal’aa*			
16	*Maganata duraa*	Dark month of the moon		
17	*Maganata Jarraa*			
18	*Maganata biritii*			
19	*Salban duraa*			
20	*Salbaan bal’aa*			
21	*Salbaan dullacha*			
22	*Gardaduma*			
23	*Sonsa*			
24	*Ruruma*			
25	*Lumasa*			
26	*Gidada*			
27	*Ruda*	

Source: RP, 2021.

Study in Botswana quoted in Loretta (2014) support this current study, disclose that the moon controlled the tides and regulate annual agricultural activities and production. It further stated that months are divided up according to the phases of the moon. The author of the ancient Greece De Signs of weather study quoted in Beardmore (2013) did sketch out a basic time-reckoning system/seasonal calendar that were based on a mixture of stellar, lunar and solar patterns. Astronomical time-reckoning which is tied to a seasonal structure, has been argued and demonstrated in works of ( Scofield, 2010). It stated that the first method, used to describe the weather for each quarter of the year, utilizes horoscopes calculated for the new or full Moon that most closely precedes the equinoxes and solstices.

### 2.3 Weather indicating physical features of the Moon

It is identified through this study that future weather indicating physical features of the Moon are categorized in to three distinctive interrelated features: 1) probable inclination/direction of the first date new moon and, 2) formation of the ring/cloud surrounding the moon, 3) Colour patterns of the moon.

Probable Direction/Inclination of the first date new moon: In connection with this there are three predetermined probable direction of the first date new moon. In this case, 1) when the probable inclination of the first date new moon of December, January and February dry periods are towards south direction; the condition of the upcoming rainfall season is comparatively more favorable to the area in the South direction than elsewhere. In this regard, according to the local community; the benchmark/the reference area of the probable south ward inclination of the first day new moon is Dire district. The upcoming season rainfall will more favorable for the *Dire* district including the area towards south of *Dire* such as *Moyale* and *Miyo* than the districts of the Borana zone in the other three direction. The inclination of the first day new moon to this direction is mainly to signals *Ganna* rainfalls (March to May). During such inclination of the moon/towards *Dire* direction; *Finna*/condition is more very suitable for cattle in all the dry land areas of Borena. In this regard, in the last *Bona Hagaya* (December to February) and *Bona Addolessa* (June to September) of 2021 the first date new moon is not inclined into the southwards as the result *Ganna* (March to May) and *Hagayya* rainfall (October to November) of 2021 was very poor in the area south of *Dirre* district of the Borana zone. This is in consistent with the study of Lawrence (2021) argued there is universal belief that the planets, solar system and their movements around the Earth affected atmospheric conditions and weather.

Similarly, 2) when the probable inclination of the first date new moon of June, July and August (during early period of the drought season known as *Bona Adolessa*) are inclined towards North direction; the condition of the upcoming rainfall season will comparatively more favorable to the area in the North direction than elsewhere. In this regard, according to the local community; the local benchmark/the reference area of the probable North ward inclination of the first date new moon is *Gomole* district. The upcoming season rainfall will more favorable for the *Gomole* district including the area towards North of *Gomole* than the other districts of the Borana zone in three directions. This phenomenon was actually happened in *Ganna* and *Hagayya* rainfall season of 2021. *Ganna* and *Hagayya* rainfall season of 2021 was more favorable to *Gomole* and area to the North of *Gomole* district of Borana zone. In the study area, the inclination of the first date new moon towards north direction is mostly used to signals *Hagaya* rainfall season. In this regard, when the inclination is made towards north direction the upcoming season/condition will be more suitable for Goat and camel than cattle in all the dry land areas of Borena zone. Contrary to this, 3) When the first new moon is emerged towards East direction/upwards during the dry season of bona *Adolessa/Bona Hagayya*; the upcoming season rainfall will be delayed or the probability of severe drought will be high. Study in Tanzania ( Elia 2014) revealed that when a half moon is constantly appearing in north direction it signals rainfall, however, it did not identified the time lapse between observing the indicating features of the moon and the occurrence of rainfall which is very mandatory in making early preparedness.

Tiny clouds surrounding the Moon: This study identified six predetermined probable direction through which tiny clouds are surrounding the moon. First) Moon is surrounded with a tiny synthesized cloud; during the dry season of the *Bona Addolessa (June to September) and Bona Hagaya* (December to February) moon is surrounded by a tiny synthesized cloud. The tiny cloud surrounding the moon is formed on the seventh date of new moon. This sign of the moon is indicating that the upcoming rainfall season will be very promising in its entire dimension i.e. rainfall will be very good in time of initiation, in amount and in physical patterns of distribution. In addition, it indicating that there will be favorable condition in the upcoming season which is locally called *Finnaa* (enough/sufficient amount of water, pasture and milk). Furthermore, it indicates that there will be no fear of risk of disease (both of human and livestock disease) outbreak and local conflict. The amount of rainfall in the upcoming season will be determined by the physical pattern/direction through which tiny cloud is formed around the moon in the night sky. In line with this, when the Moon is surrounded by tiny cloud while it is on the eastern, mid and western direction of the earth during the very early period of Bona *Adolessa*/ *Bona Hagaya* it implies that the upcoming season rainfall will be high, medium and small, respectively. This is similar with the study quoted in Beardmore, (2013) which stated that the seemingly celestial is explained by Aristotle as being firmly terrestrial. In addition, the temporal pattern of the *Danbala*/tiny cloud that is surrounding the moon has its own implication on the upcoming season rainfall. For example, when the moon is surrounded by tiny cloud in October and in April; the upcoming rainfall season of the *Ganna* (end of February up to May) and *Hagaya* (end of September up to November) would be very promising in its entire dimension, respectively. Secondly, when a tiny cloud that has a gap/a get/is surrounding the moon during the dry period of Bona Adolessa (June to August) and Bona Hagaya (December to March) will indicating that the forthcoming season (rainfall season of October to November) and rainfall of (March to May) would be very promising, respectively.

In contrast, thirdly, when a tiny cloud is not surrounding the moon during the dry season of *Bona Adolessa*/ *Bona Hagaya*/it is indicating that the upcoming rainfall season is too little/the probability of the occurrence of the prolonged drought would be very high. In addition, the moon is stunted/shirked/in physical appearance and looks like reddish in color. Fourthly, when a tiny segment of cloud is observed inside the moon during the dry season it implies that there will be normal occurrence of rainfall in the upcoming season. Fifthly, the direction on which cloud is constructed around the moon has decisive factors on the amount of rainfall of the upcoming season. When the moon is surrounded by (constructed) tiny cloud after 15 ^th^ dates of new moon while it is on the eastern, mid of the sky and western direction during the dry season it is indicating that the upcoming season rainfall will be very promising, medium and small, respectively. Furthermore, when the moon is surrounded by tiny cloud in October it indicates that the upcoming rainfall season of the *Ganna* (end of February/March up to May) will be very good in its entire dimension. Similarly, when the moon is surrounded by tiny clouds while it is in the east, mid of the sky and west in April it is indicating that the upcoming rainfall season of the *Hagaya* (October to November) will be very good in its entire dimension. In contrary to this, study ( Elia, 2014) in Tanzania argued that when the moon is surrounded by clouds, the villagers believe that rainfall will be small quantities.

Color patterns of the Moon: In the study area it is believed that when the color of the moon looks white, big, shine, cloudy and shivering during the current particular period of the dry season; it is indicating that the upcoming rainfall season will promising in its all dimension. However, when the color of the moon is dirty and stunted during the current particular period of the dry season; it is indicating that the upcoming rainfall season will be very little. This phenomenon was actually happened in *Hagayya* and *Adolessa* dry season of 2021. Study in Tanzania ( Lehoux 2021) revealed that the moon’s brightness and its haloes, taken just before and after its significant phases, give weather predictions. Similar studies ( Elia 2014) In Tanzania revealed that when farmers observe a halo of light around the moon, they believe as it signifies that the moon is surrounded by water and that this forecasts rain. Study ( Oruru *et al.*, 2016) in Uganda among the Baganda clan expect sunshine when the moon is bright red in color. However, both of these studies were not identified the specified lapse time between the observations of the indicators and occurrence of the rainfall.

### 2.4 Input of indigenous weather indicating features of the Moon in livelihood decision making

In this study it is identified that since time immemorial local community in the study area has far-fetched trust in the role of indigenous weather forecasting practices, especially, in the role of seasonal weather indicating features of the moon in drought resilience. Every daily livelihood activities: management of water and pasture land, mobility, herd splitting, herd diversification, social support and livestock selling and opportunistic crop farming are undertaken depending on the indigenous weather/drought forecasting information that are especially found through defining of weather indicating features of the moon. In addition, they commonly use and believe in its role of prediction/in disaster risk reduction such as drought, flood, disease/pest outbreak and local conflict. In this regard, forecasting based on the weather indicating features of the moon are more accurate and trusted in livelihood decision making process than the other aspects of the astronomic features and/or than the other aspects of the indigenous weather forecasting objects currently, under use in the study area. In line with this, although all members of the community (including children and women) could have basic skill of forecasting weather events; all have not equal capacity, *i.e.* there are differences among individuals in accuracy of forecasting. In line with this, individuals who have mastered in defining physical and temporal patterns of the moon in the process of forecasting future events including weathers are locally known as *Ayyaantu.* Currently, curtailments of mobility, severity of drought, weakening of traditional institution, leaving of traditional life style and death of the knowledgeable elders have challenged the potential effects of the indigenous weather forecasting indicators of the features of moon among Borana pastoralists.

Study of ancient Greece weather signs study ( Beardmore 2013) disclose that popular-practical astronomy originating far back beyond the beginnings of writing, and having gained particular traction in Babylonia, where known to have taken place in systematic observations of celestial and meteorological phenomena. It further stated that astro-meteorology as being connected to the weather; it began life bound up with time-reckoning and specific activities. In this regard ( Beardmore 2013) posited that regularities formed by the motions of celestial objects provided the necessary context upon which many structural symbolic patterns were built to regulate human activities on the earth.

## 3. Conclusion and recommendation

**Conclusion**: It is identified through this study that since time immemorial indigenous astronomic weather forecasting knowledge is the driver of livelihood decision making among Borana Oromo Pastoralists of Southern Ethiopia. In this regard, physical and temporal and/or probable predetermined day patterns of the emerging of new moon are the main features of the moon that are commonly used by the local community in the process of indicating future weather phenomena. Day patterns of the emerging of new moon is relatively more accurate and is relatively widely used than the other features of moon in weather forecasting in the study area. The prediction that is displayed via the observation of the moon would offer sufficient lapse time in a more than/within ahead of the three months lead time which is sufficient for making early preparedness in advance of the occurrence of the weather/adverse events.

**Recommendation:** Borana pastoralists have full trust in indigenous weather forecasting knowledge in leading vis-à-vis of their every daily livelihood activities. Hence, the following recommendations are mandatory in demonstrating the full potential of the indigenous knowledge weather forecasting knowledge:

1. It is better to strengthening traditional institutions in order to regain and rehabilitate the full functional capacity of the indigenous weather forecasting knowledge,
2. Borana pastoralists define outlooks of astronomic objects (sun, moon and stars), observe atmospheric characteristics (cloud, wind, temperature, rainfall and lightning), read physical condition of biotic objects (livestock, tree, wild life, birds, social insects, Reptiles etc.) and rememorize the unique and known events in each Geda cycles periodically in the process of future weather forecasting. In this regard, it is advisable to undertake detail in-depth study; and making documentation of the findings of each objects in order to preserve and use this body of knowledge in sustainable manner,
3. It is better to acknowledge and mainstreaming the indigenous weather knowledge in to formal institutional structures and integrate with science to improve its applicability and role in drought resilience.

## Data availability

### Underlying data

OSF: Underlying data for “Indigenous Astronomical Knowledge based Seasonal Weather Forecast: Evidence from Borana Oromo Pastoralists of Southern Ethiopia” https://doi.org/10.17605/OSF.IO/4K2QM; archived at https://archive.org/details/osf-registrations-4k2qm-v1

Data are available under the terms of the Creative Commons Zero “No rights reserved” data waiver (CC0 1.0 Public domain dedication).

## Footnotes

^1^
The first day on which each new moon is emerging in the night sky is locally called ayyaana ji’aa.

^2^
See remarks on the start and end of the counting of the moon dates of a month in this section.

^3^
The day on which no sign of moon is observed in the night sky.

^4^
See remarks on the start and end of the counting of the moon dates of a month in this section.

^5^
See remarks on the start and end of the counting of the moon dates of a month in this section.

^6^
See remarks on the start and end of the counting of the moon dates of a month in this section.

^7^
See remarks on the start and end of the counting of the moon dates of a month in this section.

^8^
See remarks on the start and end of the counting of the moon dates of a month in this section.

^9^
See remarks on the start and end of the counting of the moon dates of a month in this section.
