# Supplementary material for: Indigenous astronomical knowledge based seasonal weather forecast: evidence from Borana Oromo pastoralists of Southern Ethiopia
Source: F1000Res. 2022 Oct 25;11:1217. [Version 1] doi: 10.12688/f1000research.121926.1 (PMC10726095; doi:10.12688/f1000research.121926.1)
Supplement: Peer review history

# Reviewer report: Reviewer response for version 1

**DOI:** 10.5256/f1000research.133844.r211170

**Published:** 2023-12-17

**Authors:** Jarita C Holbrook

**Affiliations:** 1 Science, Technology & Innovation Studies (STIS), The University of Edinburgh, Edinburgh, Scotland, UK

This article is a positive contribution to Indigenous weatherlore using the Moon. The social context and the demarkation of the types of expertise with their local names will be helpful for future researchers and those studying other peoples within the region. The authors smartly present three uses of the Moon: 1) on which day the new moon/first crescent falls 2) if and when clouds appear around the moon and 3) the appearance of the moon in terms of colour and 'shivering' (scintillation). There is always the issue of providing more common references (i.e. Gregorian Calendar Months) to understand lunar systems. This may be solved by referencing to other 13 month lunar calendars instead. It is significant that such complicated Indigenous logics are presented in the African context. Of interest is if there are associated tools or artefacts that aid in doing the calendar tasks correctly. The authors present the internal logic of whether the predictions were successful or not, this could be followed up by presenting the rainfall record for the region for 2021. 

 There are significant and distracting grammatical errors, thus this article could be improved by having a final grammar edit.

Is the work clearly and accurately presented and does it cite the current literature?

Partly

If applicable, is the statistical analysis and its interpretation appropriate?

Yes

Are all the source data underlying the results available to ensure full reproducibility?

Yes

Is the study design appropriate and is the work technically sound?

Yes

Are the conclusions drawn adequately supported by the results?

Yes

Are sufficient details of methods and analysis provided to allow replication by others?

Yes

Reviewer Expertise:

Cultural Astronomy, African Indigenous Astronomy, Science & Technology Studies

I confirm that I have read this submission and believe that I have an appropriate level of expertise to confirm that it is of an acceptable scientific standard.

# Reviewer report: Reviewer response for version 1

**DOI:** 10.5256/f1000research.133844.r211165

**Published:** 2023-11-01

**Authors:** Duane Hamacher

**Affiliations:** 1 The University of Melbourne, Melbourne, Victoria, Australia

This detailed paper dives into the astronomical knowledge of the Borana Oromo pastoralists of Ethiopia, with a focus on lunar calendar systems. It provides a useful and detailed ethnographic analysis of lunar calendars, focusing on a culture that has relatively little published about its star knowledge.

 The paper goes into some depth, with a well-laid-out focus on lunar calendars and their link to seasonal calendars and weather forecasting, providing a substantial amount of detail and does so within the local cultural framework.

 Particular interest is given to forecasting techniques using the moon, such as haloes, cusp orientation (inclination of the Moon), clouds, and lunar location on the celestial dome. This is one of - if not the most - detailed studies of Indigenous lunar calendars and lunar astronomy in the literature. It represents an invaluable contribution to the study of cultural/Indigenous astronomy and presents a useful case study in cultural science focused ethnography.

Is the work clearly and accurately presented and does it cite the current literature?

Yes

If applicable, is the statistical analysis and its interpretation appropriate?

Yes

Are all the source data underlying the results available to ensure full reproducibility?

Yes

Is the study design appropriate and is the work technically sound?

Yes

Are the conclusions drawn adequately supported by the results?

Yes

Are sufficient details of methods and analysis provided to allow replication by others?

Yes

Reviewer Expertise:

Cultural astronomy, Indigenous astronomy, Indigenous Science, Indigenous Knowledge, history and philosophy of science, astrophysics, ethnography

I confirm that I have read this submission and believe that I have an appropriate level of expertise to confirm that it is of an acceptable scientific standard.

# Reviewer report: Reviewer response for version 1

**DOI:** 10.5256/f1000research.133844.r211167

**Published:** 2023-10-24

**Authors:** Hilding Neilson

**Affiliations:** 1 Physics & Physical Oceanography, Memorial University of Newfoundland, St. John's, Newfoundland and Labrador, Canada

The article is a valuable discussion of Indigenous knowledges using the Lunar phases as a method for weather prediction. The authors demonstrate and reproduce local Indigenous knowledge for science. This work is important and I urge the journal to accept the article after revisions.

 Some comments:

- There are grammar mistakes in the article that would benefit from editing.
- There is a lot of information in the Tables indicating temporal patterns that could be represented by a plot or a few plots.
  The plots could illustrate the days that are double-counted, etc.
- The authors discuss the 12 moons but since the time from New Moon to New Moon is 29.5 days then some years there will be a 13th Moon. How does this fit into the local knowledges?
- Similarly, the timing of a Moon will vary throughout the month depending on the lunar cycle. That is, sometimes, New Moon will be in the beginning of a calendar month and sometimes in the middle of the month. How does this impact the description? By and large, this is not an issue with methodology, but defining the Moon as January Moon of March Moon could confuse the reader, perhaps it would be clearer if the authors write the Moon as the first Moon of the year, second Moon and so on.
- The authors are inconsistent with using a capital 'I' and small 'i' when describing Indigenous peoples, methods, etc.

Is the work clearly and accurately presented and does it cite the current literature?

Yes

If applicable, is the statistical analysis and its interpretation appropriate?

Not applicable

Are all the source data underlying the results available to ensure full reproducibility?

Partly

Is the study design appropriate and is the work technically sound?

Yes

Are the conclusions drawn adequately supported by the results?

Partly

Are sufficient details of methods and analysis provided to allow replication by others?

Partly

Reviewer Expertise:

Astronomy, Indigenous methodologies

I confirm that I have read this submission and believe that I have an appropriate level of expertise to confirm that it is of an acceptable scientific standard, however I have significant reservations, as outlined above.
